# Comparison of commercial ELISA assays for quantification of corticosterone in serum

**DOI:** 10.1038/s41598-017-06006-4

**Published:** 2017-07-27

**Authors:** Anne Marie Kinn Rød, Nina Harkestad, Finn Konow Jellestad, Robert Murison

**Affiliations:** Department of Biological and Medical Psychology, University of Bergen, Bergen, Norway

## Abstract

Enzyme-linked immunosorbent assay (ELISA) kits are widely used to quantify corticosterone levels for the assessment of stress in laboratory animals. The aim of this experiment was simply to evaluate if four different and widely used commercial ELISA assays would yield the same or similar values of corticosterone in serum samples taken from laboratory rats after the mild stress of being held for sampling blood from the saphenous vein. Trunk blood was sampled from 32 male Wistar rats 30 minutes after this mild stress exposure and analysed with each of four commercial ELISA kits. Both the Arbor Assays and the DRG-4164 kits were significantly higher than the DRG-5186 and the Enzo kits. There were no significant differences between the DRG-5186 and Enzo kits. Overall the correlations between kits were high. In conclusion, the commercial ELISA kits tested in the present experiment yielded different values of total corticosterone in the same serum samples. The precision in determining true values of the corticosterone level is low for these commercial ELISA kits, although they may be used to determine relative differences within studies.

## Introduction

In vertebrates, a central component of the stress response is activation of the hypothalamic-pituitary-adrenal (HPA) axis. Activation of the HPA axis comprises release of corticotropin-releasing hormone (CRH), adrenocorticotropic hormone (ACTH) and glucocorticoids (GCs). The main glucocorticoid in humans is cortisol and corticosterone in rats and mice. Approximately 95% of secreted cortisol is bound to carrier proteins in the blood; 80–90% to Corticosteroid-binding globulin (CBG) and 10–15% to albumin^1^. The remaining 5% is free cortisol. According to the “free hormone hypothesis” only free GCs are considered to be biologically active^2^. It is essential to know whether a particular analytic method measures free or total cortisol/corticosterone in blood.

The most common methods for quantification of hormones such as cortisol and corticosterone are competitive binding immunoassays i.e. radioimmunoassay (RIA) and enzyme-linked immunosorbent assay (ELISA). Previously RIA was the most extensively used method, but requires the use of radioactive isotopes, specialized equipment and strict routines for the handling of radioactive materials. More recently, an increased awareness of the health risks associated with working with radioactive materials, along with stronger protective legislation, has reduced the use of RIA. The development of commercial ELISA kits has allowed quantification of hormones without the use of radioactivity, and today they are widely used for the assessment of stress in laboratory animals. However, to our knowledge, no comparative studies have been published to show how different commercial ELISA kits quantify the same amount of cortisol or corticosterone in identical samples from rats.

The aim of this experiment was simply to evaluate if four different and widely used commercial ELISA assays would yield similar values of corticosterone in the same serum samples taken from laboratory rats after a mild stress induced by being held for blood sampling from the saphenous vein in the hind leg.

## Methods

### Ethical considerations

The experiment described in the present work has been approved and registered by the Norwegian Animal Research Authority (Permit Number: 20146661), and conducted in accordance with Norwegian law and the European animal convention (1986).

To comply with one of the 3R’s (Reduction), animals in the present project were re-used from a project with research questions about housing conditions. The protocol that animals were exposed to during the first 7 weeks in that experiment is summarized below.

### Animals and housing

32 male rats (7 weeks old, Wistar HanTac, Taconic, Denmark) were single housed in conventional (CON; n = 16; 189.50 ± 6.67 g) or individually ventilated cages (IVC; n = 16; 189.25 ± 7.37 g). All rats were kept in the same room with a 12:12 hour light/dark schedule (lights on at 07:00 h and one hour progressive increase and dimming). The cages were polypropylene Euro-standard Type III H cages (425 × 266 × 185 mm: floor area 800 cm^2^). IVC cages, with polypropylene lids had 75 air shifts per hour. Average cage temperature was 22 °C and average relative humidity was 65%. The rats had free access to food and water. Upon arrival, rats were randomly placed in either IVC or CON cages stratified by weight.

### Protocol

During the first 7 weeks rats were kept in the animal room and only handled during weighing, cage change and behavioural testing (12 min open field test and 1 hour water avoidance test). During week 8, all rats were handled for 2 minutes each day for 4 days. During handling the rats were habituated to the holding procedure later to be used for blood sampling. This consisted of wrapping the rat in a towel and one hind leg was pulled out and held still. On the day before blood sampling, the hind legs of the rats were shaved. On the experimental day the rats were moved one by one in their home cage to an adjacent room. Each rat was held in a towel, stasis was applied to the saphenous vein and the vein was punctured by a needle. Blood was collected in serum tubes. The time duration of the whole process from taking the rat out of the rack to completed blood sampling was 2–3 minutes. Data from these saphenous vein blood samples are not included in the current analyses.

### Blood collection for corticosterone quantification

30 minutes after holding the rats for blood sampling from the hind leg, the rats were anesthetized with Isoflurane and decapitated. Trunk blood was collected in serum tubes. Blood sampling took place between 09:00 and 12:00 h.

### Sample preparation and corticosterone assays

Trunk blood was allowed to coagulate at room temperature in serum tubes for 1 hour. Samples were centrifuged in an Eppendorf 5415R centrifuge at 10 000 rpm for 10 minutes. Serum was aliquoted and frozen at −20 °C until analysis. Disposable plastic pipette tips were used for aliquotation of samples and for all further analyses (Thermo Scientific, F1-Clip Tip Pipeting system). Each serum sample (n = 32) was analysed in duplicate with each of the four ELISA kits: DRG EIA-5186 (DRG Diagnostics, Marburg, Germany), DRG EIA-4164 (DRG Diagnostics, Marburg, Germany), Enzo ADI-900-097 (Enzo Life Sciences, Plymoth meeting, PA, USA) and Arbor Assays K014-H1(DetectX^®^, Arbor Assays™, Eisenhower Place Ann Arbor, MI, USA) according to the manufacturers‘ instructions. The absorbance at each assay was read at a wavelength of 405 nm with a plate reader (Wallac 1420 Multilabel counter, PerkinElmer, Norway). Based on standard curves run in duplicate on each plate, corticosterone concentration was determined with the aid of Workout 2.5 software (DAZDAQ LTD., East Sussex, England).

The *DRG-5186* kit for corticosterone is produced specifically to analyse rat and mouse serum and plasma samples. Wells are coated with polyclonal rabbit anti-corticosterone antibody (rabbit immunized with commercially produced corticosterone). See Table 1 for more information about the kit.

**Table 1:** Information from manufacturers kit manual and included in the kits.

	DRG-5186	DRG-4164	Enzo	Arbor Assays
Standard series:	15–2250 ng/ml.	1.7–83 ng/ml	0.032–20 ng/ml.	0.078–10 ng/ml
Analytical sensitivity:	4.1 ng/ml	0.5647 ng/ml	0.02699 ng/ml	0.0186 ng/ml
Amount of sample (for dilution):	20 µl	10 µl	10 µl	5 µl
Sample dilution:	1:1	1:10	1:40	1:100
Release from CBG:	n.s.	n.s.	YES	YES
Internal quality control	Yes*	Yes	No	No
Cross reactivity (%):				
Progesterone	0.7	7.4	1.7	0.24
Aldosterone	0.2	n.a.	0.18	0.62
Cortisol	0.3	0.3	0.046	0.38
11-Deoxycorticosterone	2.4	3.4	28.6	12.3
Dehydroepiandrosterone	n.d.	n.a.	n.a.	n.a.
Estriol	n.d.	n.a.	n.a.	n.a.
Estradiol	n.d.	n.a.	n.a.	<0.08
17-hydroxyprogesterone	n.d.	n.a.	n.a.	n.a.
18-Hydroxydeoxycorticosterone	n.d.	n.a.	n.a.	n.a.
Testosterone	n.d.	n.a.	0.13	n.a.
Pregnolone Prognenolone	n.d.	0.3	<0.03	n.a.
11-Dehydrocorticosterone	n.a.	1.6	n.a.	n.a.
Tetrahydrocorticosterone	n.a.	n.a.	0.28	0.76
β-Estradiol	n.a.	n.a.	<0.03	n.a.
Cortisone	n.a.	n.a.	<0.03	<0.08
11-dehydrocorticosterone acetate	n.a.	n.a.	<0.03	n.a.
Dexamethasone	n.a.	n.a.	n.a.	0.12
Corticosterone-21-Hemisuccinate	n.a.	n.a.	n.a.	<0.1
Other steroids	n.a.	<0.1	n.a.	n.a.

*Recommended quality control kit (DRG CTL-5262). CBG -Corticosteroid-binding globulin; n.s. not specified in the manual; n.d. cross reactivity not detected for these substances; n.a. the cross reactivity is not assessed for these substances.

The *DRG-4164* kit was originally produced to analyse corticosterone in humans. Wells are coated with polyclonal anti-corticosterone antibody (polyclonal antibody from rabbit). See Table 1 for more information about the kit.

In the *Enzo* kit, wells are coated with donkey antibody specific to sheep immunoglobulin G (IgG). The Enzo assay was provided with a ‘steroid displacement reagent’ (SDR) to release corticosterone bound to CBGs. Thus, the Enzo assay explicitly quantifies the total amount of corticosterone in the samples. See Table 1 for more information about the kit.

In the *Arbor Assays* kit, wells were coated with donkey anti-sheep IgG. The kit was provided with a ‘Dissociation Reagent’ to release corticosterone bound to CBGs. Thus, it quantifies the total amount of corticosterone. See Table 1 for more information about the kit.

For internal quality control of the DRG-5164 kit analysis (to assess intra-assay variation), the recommended quality control kit (DRG CTL-5262) was used. The quality control kit included high and low assay control samples, and established acceptable ranges were stated in the manual. Results from the assay control samples must be within the established acceptable range to verify proper performance of the ELISA analysis. In the DRG-4164 kit, high and low assay control samples and established acceptable ranges were provided for internal quality control. Neither the Enzo kit nor the Arbor Assays kit were provided with internal quality controls (high or low assay control), nor were such internal quality controls suggested in the kit manuals.

### Statistics

Serum samples and standards were analysed in duplicate for each kit and measures were averaged in the plate reader software described above. Statistical analyses were performed using Statistica 13.0 (Dell Inc.) software. Results are expressed in mean ± Std.dev. Due to significant heterogeneity of variance non-parametric statistical analyses were applied. The potential effects of housing conditions were controlled for by comparing housing conditions by the Mann-Whitney U test. To assess differences in corticosterone levels provided by the four the ELISA kits, the Kruskal-Wallis test was used. Correlations between the four assays were tested by Spearman Rank Order Correlations.

To evaluate internal quality control (intra-assay variation), the coefficient of variation (CV%) was calculated from the mean and standard deviation of multiple replicates of high and low assay controls.

## Results

The Enzo and Arbor Assays kits measure total corticosterone, as these kits are provided with substances to release corticosterone bound to CBGs. This is clearly stated in the kit manual. This information was lacking in both the DRG kit manuals.

No significant housing effects were found within each kit and thus the housing variable was not included in further statistical comparisons.

**Figure 1:**
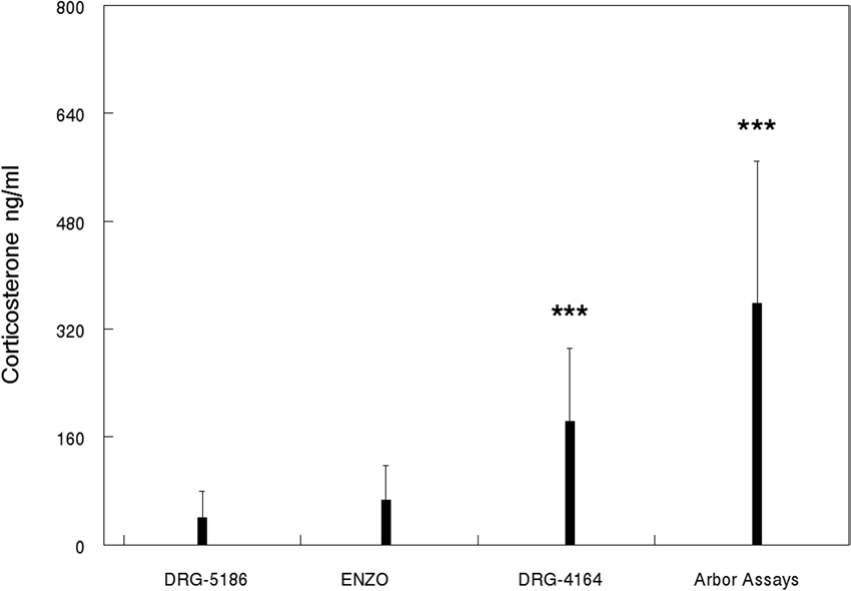
Corticosterone (ng/ml) measured in the four ELISA assays. Bars and whiskers indicate mean + Std.dev. ***p < 0.001 difference from DRG-5186 and from Enzo (Kruskal-Wallis test).

The Kruskal-Wallis test showed a significant difference between kits (H(3, N = 128) = 84.66, p = 0.000). The Arbor Assays kit yielded the highest mean corticosterone values and the largest variance (357.75 ± 210.52). This was significantly higher than both the DRG-5186 (40.25 ± 39.81) and the Enzo kit (66.27 ± 51.48), p = 0.0000 for both comparisons and close to significantly higher than the DRG-4156 kit (183.48 ± 108.02, p = 0.0596). The DRG-4164 assay showed significantly higher corticosterone values compared to the Enzo and DRG-5186 kits (p’s < 0.0004 and 0.0000 respectively). There was no significant difference between corticosterone values measured by the ENZO kit and the DRG-5186 kit. See Fig. 1.

Spearman Rank Order Correlations showed that all results from the various assay kits (n = 32 for each assay) were positively correlated (p’s < 0.05): DRG-5186 vs Enzo (r = 0.95); DRG-5186 vs DRG-4164 (r = 0.95); DRG-5186 and Arbor Assays (r = 0.88); Enzo vs DRG-4164 assay (r = 0.93); Enzo vs Arbor Assays (r = 0.84) and DRG-4164 vs Arbor Assays (r = 0.84).

### Internal quality control

For both DRG kits, the high and low assay controls were within the established acceptable ranges stated in the manual. The intra-assay coefficient of variation (CV) for the DRG EIA-4164 was 15.5% for the low assay control and 6.8% for the high assay control. For the DRG-5186 kit CV was 14.9% for the low assay control and 4.1% for the high assay control. No internal quality controls for evaluation of intra-assay variation were included or suggested by the manufacturers of the Enzo and Arbor Assays kits.

## Discussion

The commercial ELISA kits tested in the present experiment yielded different values of corticosterone in the same serum samples. Both the Arbor Assays and the DRG-4164 kits were significantly higher than the DRG-5186 and the Enzo kits. There were no significant differences between the DRG-5186 and Enzo kits. Overall the correlations between kits are high, but the Arbor Assays kit shows the weakest correlations with the Enzo and the DRG-4164 kits.

There was a large variation between corticosterone levels 30 min after holding the rats for blood sampling. See Fig. 1. The relatively large variation between animals within each kit cannot be explained by the two different housing conditions, since there were no significant effects of housing condition detected by any of the kits. The high correlation between kits demonstrate that animals with high corticosterone response measured by one kit are also highly likely to be measured as a high responder by other kits. The large difference in variation between kits as shown by significant heterogeneity of variance may be explained by the fact that the kits contain different capture antibodies and different substances to release corticosterone bound to CBGs. They also have different analytical sensitivity and report various cross reactivities to substances other than corticosterone (see Table 1). All these differences between kits may contribute to the large difference in values of total corticosterone measured in the same serum samples.

From the analytic method used in the present study it is impossible to know if the true corticosterone values are as high as analysed in the DRG-4164 and the Arbor Assays kits or as low as analysed in the DRG-5186 and the Enzo kit. High results may be due to more complete release of corticosterone from CBG or to binding unspecific epitopes on the capture antibodies. According to the manufacturers’ documentation, the Enzo and the Arbor Assays kits have been tested for cross reactivity to more hormones than have the DRG kits. However other proteins in the serum sample may bind to the capture antibodies and thus give high results. Low results may be due to incomplete release of corticosterone from CGB. The different CBG releasing substances provided with the kits may release different fractions of corticosterone from the CBG, possibly due to different dilution of the releasing agent relative to the sample or due to different releasing properties of the agent itself. In the kit manuals the fraction of released corticosterone from CBG is not addressed, and scarce information about the releasing agent is given. Additional research is needed to resolve this important aspect about the fraction of released corticosterone from CBG in each kit.

It is essential for the researcher to know whether the kit is measuring free or total (free and bound) corticosterone. For some kits used in this experiment the manuals do not provide the customer with sufficient information. The manual for the Enzo and Arbor Assays kits clearly state that total corticosterone is measured, as these kits are provided with substances to release corticosterone bound to CBGs. However, in the DRG kits’ manuals this was not clearly specified. According to the manufacturer through e-mail correspondence, the incubation buffer used in the DRG-5186 assay releases bound corticosterone, indicating that the assay quantifies total corticosterone concentration in the samples. Even if these kits are provided with corticosterone releasing agents the fraction of released corticosterone is not clear, as discussed above. The manufacturer of the DRG-4164 assay also informed us that their kit quantifies the total corticosterone concentration in the samples, but in what way was not explained to us in the correspondence. For incubation with the sample during assay analysis there are no specified substances added to release corticosterone from CBG in this kit. We therefore assume that the capture antibodies coated in the wells both bind free and bound corticosterone, but which fraction of corticosterone is bound in the wells is unclear also in this kit. When we asked about more information about the antibodies, we were told that it was classified. Whether a kit measures free or total corticosterone should always be clearly stated in all manuals by all manufacturers, but this is not the case for all commercial kits available today.

The DRG-5186 kit is especially made for measuring corticosterone in rats as the standards contain rat serum to minimize matrix differences between samples and standards (additional information provided by the manufacturer, but not specified in the manual). In that respect this is a valid kit. However, in studies where resting levels of corticosterone in rats are of interest, sensitivity might be too low for this kit, yielding uncertain results at the lower end of the scale. In studies where a corticosterone level below the analytical sensitivity of the DRG-5186 is expected, alternative kits should be chosen.

In papers on the quantification of corticosterone the manufacturer and product number is normally stated. Through correspondence with several manufacturers, we have learnt that some kits are identical even if they are from different manufacturers and even if the product code varies. This is due to merging of companies and production of kits in the same factory, but the company puts its own trade name on it. Some years ago, Assay Designs was fully integrated into Enzo life science and the old product name of the kit was preserved. Thus, the Enzo ADI-900-097 is identical to the kits previously reported as enzyme immunoassay kit (Assay Designs, Inc., Ann Arbor, MI, USA).The DRG-5186 kit is identical to a kit provided by Demeditec (DEV9922, Demeditec, Kiel-Wellsee, Germany). The DRG-4164 is identical to a kit provided by IBL (RE52211, IBL International GmbH, Hamburg, Germany). When comparing corticosterone results with results from other studies, this information could be useful. However the manufacturers would probably not be motivated in providing this information for commercial reasons.

Internal quality controls (QC) are important for evaluation of ELISA assay analysis. In both the kits from DRG, high and low assay controls were included or recommended for evaluation of the intra-assay variation, and provide a measure of reproducibility between wells within an assay plate. The assay controls were within the recommended range from the manufacturer. The CV for both the DRG high and low assay controls in the current experiment were below 20%. A CV below 20% is considered as a satisfactory level within ELISA assay analysis^3–5^. The inclusion of such QC is an advantage for the DRG kits over the other kits. All kits should include commercial QC for the substance in question.

Recently a similar study was published on comparison of commercial ELISA kits for the quantification of corticosterone in mice^6^. As in the present study, the authors found that different kits yielded diverse levels of corticosterone in identical serum samples and in identical faecal samples. However, they showed that the ELISA kits could accurately determine relative differences between samples from undisturbed mice and mice which were injected with adrenocorticotropic releasing hormone as a HPA axis challenge.

In conclusion, the commercial ELISA kits tested in the present experiment yielded different values of total corticosterone in the same serum samples from rats. The precision in determining the true value of the corticosterone level is low for these commercial ELISA kits. For comparisons of corticosterone levels between studies, it is essential to know which kit was used. Although exact levels of corticosterone are difficult or impossible to determine, these kits may be used to determine relative differences within studies. One should be aware of the fact that “total corticosterone” as some manufacturers state, may not actually represent the total amount of corticosterone. Additional research is needed to resolve the issue about which fraction of corticosterone is released from CBG in each kit.

## Electronic supplementary material

Supplementary Information
